# Development of a novel laboratory photodynamic therapy device: automated multi-mode LED system for optimum well-plate irradiation

**DOI:** 10.1007/s10103-024-04083-2

**Published:** 2024-05-16

**Authors:** Mustafa Zahid Yildiz, Ali Furkan Kamanli, Gamze Güney Eskiler, Haşim Özgür Tabakoğlu, Muhammed Ali Pala, Ayla Eren Özdemir

**Affiliations:** 1 Faculty of Technology, Electrical-Electronics Engineering, Sakarya University of Applied Sciences, Serdivan, Turkey; 2 Present Address: Biomedical Technologies Application and Research Center (Biyotam), Sakarya University of Applied Sciences, Serdivan, Turkey; 3 Faculty of Medicine, Sakarya University, Serdivan, Turkey; 4 Biomedical Engineering, Bakircay University, Izmir, Turkey; 5 Health Services Vocational School, Sakarya University, Serdivan, Sakarya Turkey

**Keywords:** Photodynamic therapy, Automated experiment, Dosage control, Dosimetry, Embedded systems

## Abstract

Photodynamic therapy (PDT) is a targeted treatment method that utilizes a photosensitizer (PS) to induce cytotoxicity in malignant and non-malignant tumors. Optimization of PDT requires investigation of the selectivity of PS for the target tissues, irradiating light source, irradiation wavelengths, fluence rate, fluence, illumination mode, and overall treatment plan. In this study, we developed the Multi-mode Automatized Well-plate PDT LED Laboratory Irradiation System (MAWPLIS), an innovative device that automates time-consuming well plate light dosage/PS dose measurement experiment. The careful control of LED current and temperature stabilization in the LED module allowed the system to achieve high optical output stability. The MAWPLIS was designed by integrating a 3-axis moving system and motion controller, a quick-switching LED controller unit equipped with interchangeable LED modules capable of employing multiple wavelengths, and a TEC system. The proposed system achieved high optical output stability (1 mW) within the range of 0-500 mW, high wavelength stability (5 nm) at 635 nm, and high temperature stability (0.2 °C) across all radiation modes. The system’s validation involved in vitro analysis using 5-ALA across varying concentrations, incubation periods, light exposures, and wavelengths in HT-29 colon cancer and WI-38 human lung fibroblast cell lines. Specifically, a combination of 405 nm and 635 nm wavelengths was selected to demonstrate enhanced strategies for colon cancer cell eradication and system validation. The MAWPLIS system represents a significant advancement in photodynamic therapy (PDT) research, offering automation and standardization of time-intensive experiments, high stability and precision, and improved PDT efficacy through dual-wavelength integration.

## Highlights

Multi-mode Automated Well-plate PDT LED Laboratory Irradiation System described.

Automates and standardizes time-consuming experiments.

LED wavelength and temperature stabilized for highly reproducible irradiations.

Efficacy demonstrated in 5-aminolevulinic acid (5-ALA) treatments of HT-29 colon cancer cells and WI-38 human lung fibroblasts.

## Introduction

Photodynamic therapy (PDT) is a promising approach for selectively targeting and eliminating cancer cells by activating photosensitizers (PS) using light sources. Among the available light sources, including lasers, halogen, xenon, and light-emitting diodes (LEDs), LED technology has gained considerable attention due to its versatility and potential for tailored clinical applications [1–14].

Optimization of treatment parameters such as irradiance, fluence rate, wavelength, and illumination mode is critical for PDT success [15, 16]. LED PDT has the advantage of fine-tuning light factors and improving therapy parameters, making it a promising option for a variety of clinical applications. The absorption spectrum of the photosensitizer affects the wavelength selection.

In photodynamic treatment (PDT), selecting the right photosensitizer (PS) is crucial, especially when it comes to the material’s compatibility with laser spectroscopy or laser ablation methods. A good PS should have ideal fluorescence characteristics that allow for effective excitation upon laser irradiation, letting great sensitivity and precision in subsequent detection or ablation procedures [17]. By designing an LED system, parameters which include light intensity, wavelength stability, and narrow spectrum distribution are crucial [1–5].

LED light can be delivered in continuous or pulsed mode and covers a wide wavelength range from 400 to 1200 nm [16, 18], offering potential therapeutic benefits. LED PDT has shown remarkable biological effects, including the stimulation of various cell types, modulation of cell oxidation, anti-inflammatory effects, stimulation of angiogenesis and blood flow, and antibacterial activity [16, 19–25]. These findings highlight the promise of LED-based therapy in a variety of clinical applications. LED technology, when combined with thorough temperature and current control systems, allows for exact light delivery within the specified wavelength range [26, 27]. Semiconductor technology enables highly targeted and specific delivery of light in the visible to infrared range, offering advantages in PDT applications.

However, using LEDs for PDT presents certain design challenges. Maintaining LEDs at the desired temperature is crucial for achieving the desired illumination wavelength. Thermoelectric cooling (TEC) systems with closed-loop control techniques can ensure precise temperature control for optimal LED performance [3, 27, 28]. Additionally, modifications in the illumination mode are necessary to reduce irreversible thermal damage and increase oxygen concentration in the target tissue by giving the needed time to reoxygenation, mitigating the risk of thermal damage to both the LED system and the tissue [29–31]. To maximize the effectiveness of PDT, it is crucial to develop an ideal light source system that limits thermal damage caused by excessive optical energy accumulation while ensuring adequate oxygen supply for successful therapy [31–35]. By addressing these challenges, LED-based PDT holds great promise for advancing cancer treatment.

In this study, we present the development of the Multi-mode Automatized Well-plate PDT LED Laboratory Irradiation System (MAWPLIS), a unique technology that, among PDT LED systems described in the literature, enables the automation of laborious well plate light dosage/PS dose measurement tests. The MAWPLIS system contains three separate radiation modes that allow for the desired light dose. The suggested system achieves excellent optical output stability and wavelength stability through the use of precise LED current regulation and temperature stabilization. The system specifically accomplished strong optical output stability (± 1 mW) in the range of 0-500 mW, high wavelength stability (± 5 nm) at 635 nm, and high temperature stability (± 0.2 ^0^C) in all radiation modes.

The MAWPLIS system is composed of three primary components: a TEC system, a rapid switching LED controller unit with interchangeable LED modules for various wavelengths, and a 3-axis movement system and motion controller. We investigated the optical wavelength variability and stability, optical power output, and laser resonator temperature in order to assess the system’s performance for optical radiation modes. The study focused on using 5-ALA and testing different concentrations, incubation times, light doses, and wavelengths. The HT-29 colon cancer cell line and WI-38 human lung fibroblast cell line were utilized. The combination of two wavelengths, 405 nm and 635 nm, commonly used for 5-ALA, was chosen to demonstrate effective strategies for eliminating colon cancer cells and validating the system. The MAWPLIS system offers automation, reduces user errors, and provides high stability and accuracy. This system serves as a valuable tool for the development and validation of PDT LED systems.

## Materials and methods

Four different sub-systems were used in the development of the project. The systems are the TEC system, movement system, illumination mode-appropriate LED current driver and LED illumination probe. The LED driver is designed with components that are effective at driving current for radiation modes.

### Sub-units of MAWPLIS

**Fig. 1:**
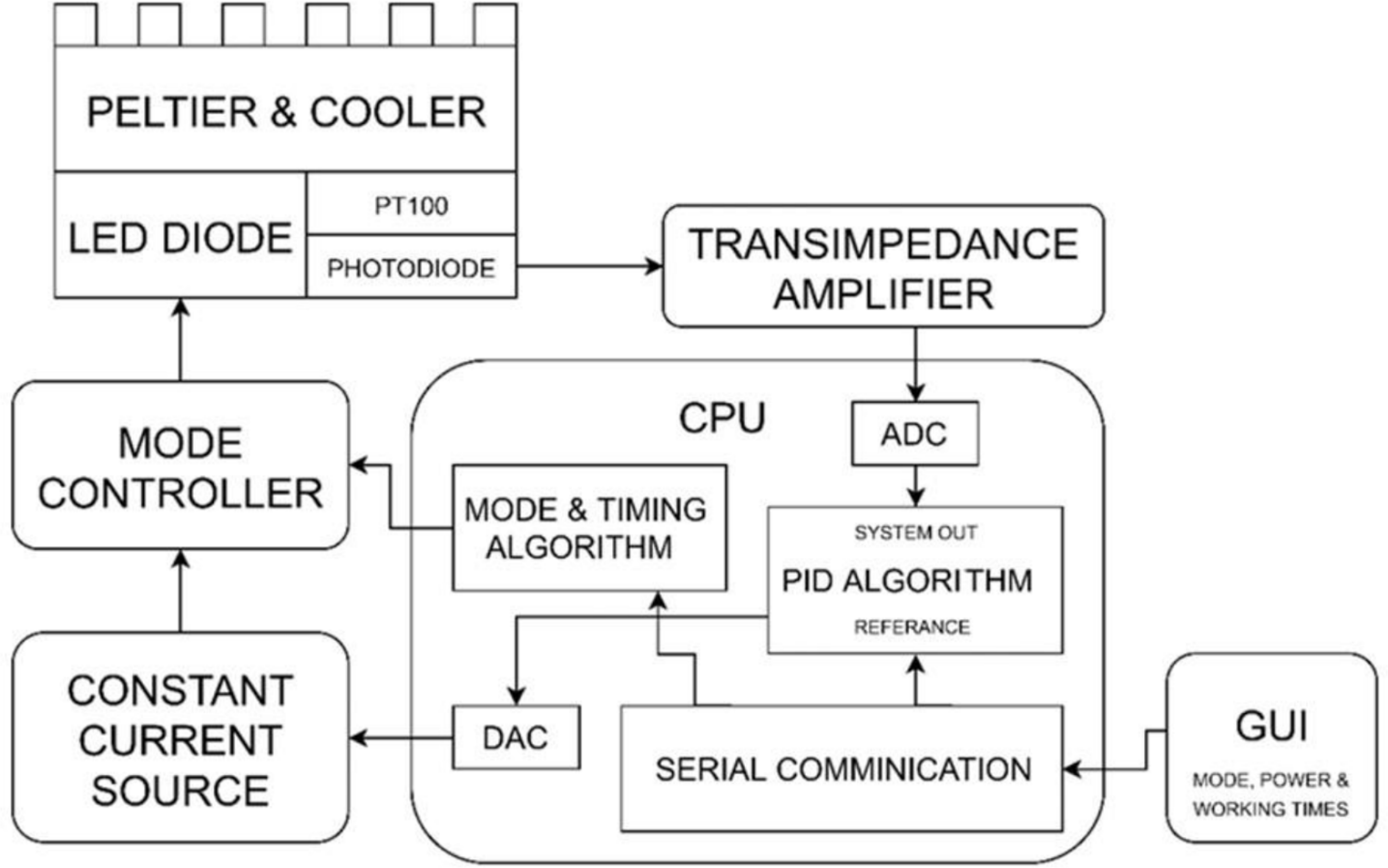
The system electronic block diagram

The LED current control mode varies based on the lighting modes used, necessitating adaption of the TEC (Thermoelectric Cooler) system to account for these changes. The second step entails creating a TEC system for wavelength stabilization and temperature stability on the LED chip. A three axis movement system for independently lighting each well on the well plate makes up the third component. To avoid wavelength shift and optic power fluctuations in LED light source illumination, the fourth component is an LED illumination probe and feedback device. The user interface for adjusting the lighting mode, duration, and other illumination-related parameters makes up the fifth component. Electronic block diagram of the system is shown in Fig. 1.

#### TEC controller

A TEC system consists of a fan, heatsink, and peltier element. The peltier element’s hot side is in direct contact with the heatsink, which is connected to the fan by heatpipes. The system’s LED is in direct touch with the peltier element’s cold side. TEC systems require highly accurate current regulation. Changing the magnitude and direction of the current enables the conversion between heating and cooling, as well as adjustments to the quantity of heat being absorbed or released. The ambient temperature has an impact on the semiconductor’s cooling capability. Therefore, the temperature of the TEC module was continuously monitored, and PID control was used to maintain the temperature of the LEDs. The setting was changed to 44 ^0^C. Peltier system was shown in Fig. 1.

#### LED driver

**Fig. 2:**
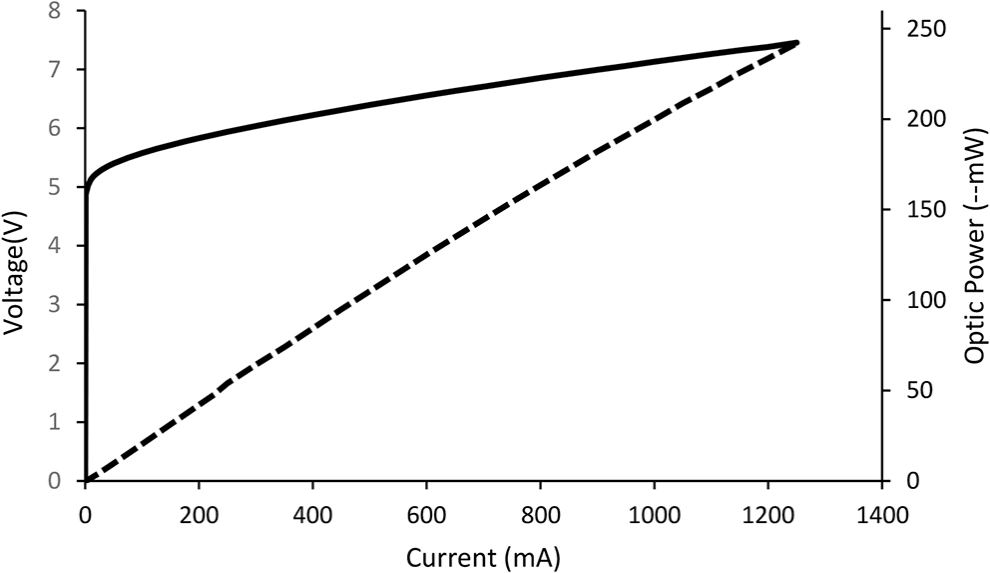
LED voltage, current and optical power. Relationship between LED current and optical power is represented by the equation y = 0.005833x, while the relationship between LED current and voltage is described by the equation y = 0.00166667x + 5

The relationship between the terminal voltages and the current flowing through the diode in LEDs is exponential. Even a minor 10% change in the LED’s voltage can cause a significant 100% change in the LED diode current. Constant current sources should be employed in semiconductor-based lighting elements for accurate optical power management. Figure 2 shows the relationship between LED current and voltage.

#### LED illumination probe and optic feedback system

**Fig. 3:**
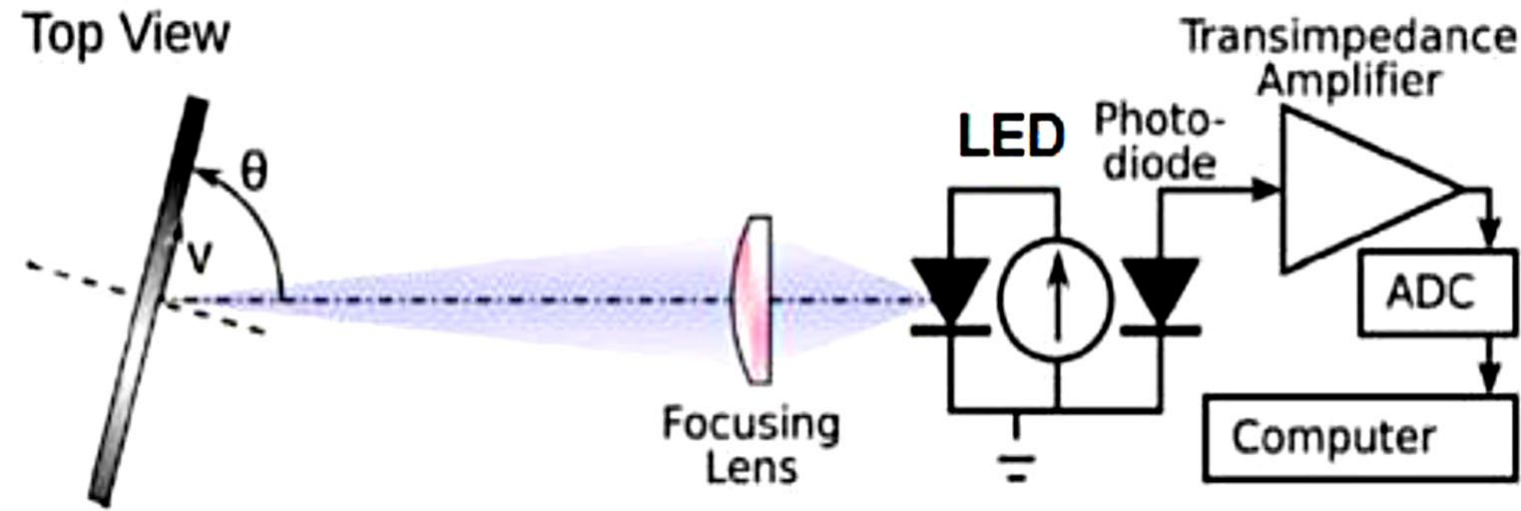
LED illumination probe and optic feedback system design

**Fig. 4:**
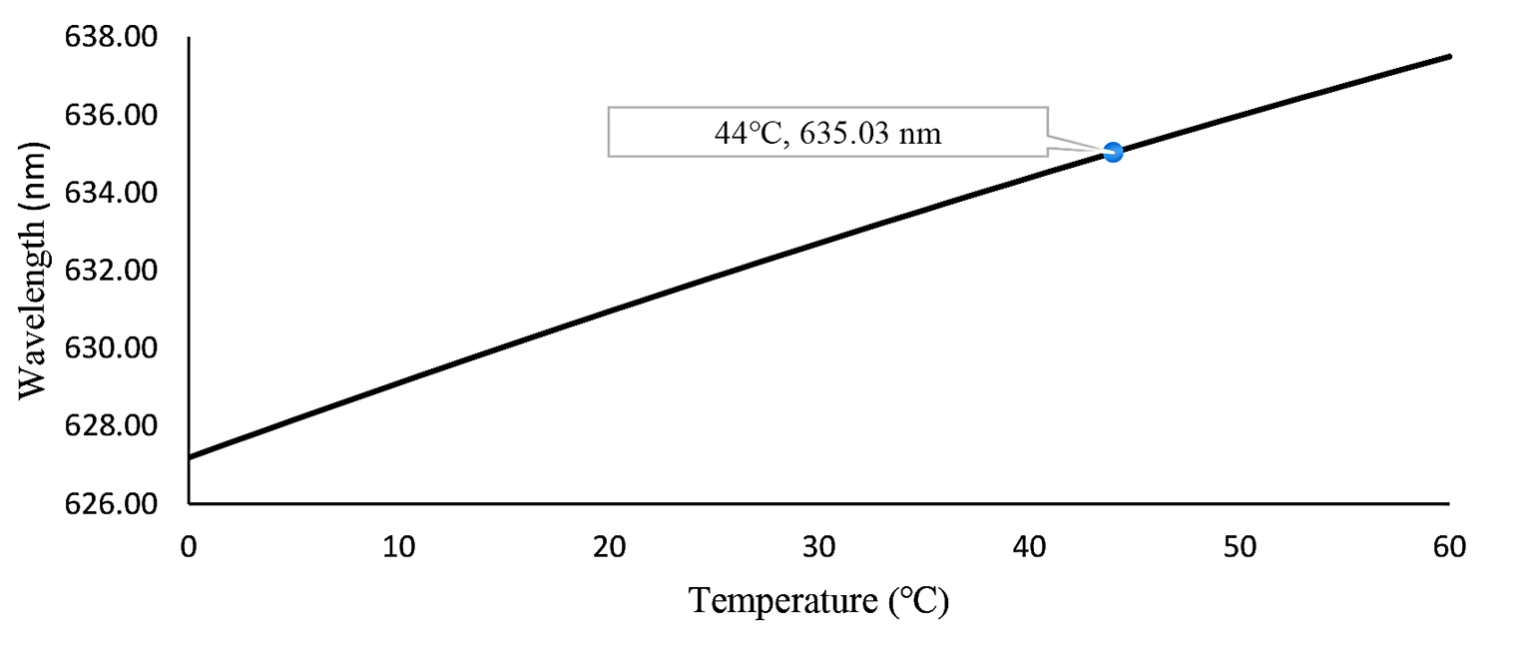
LED Junction temperature effect on LEDs wavelength

The led probe was designed to illuminate one well at a time in order to keep light from escaping and lighting up the other well. The optical power can be accurately sent to the target well with a specially built LED probe. The photodiode-equipped probe is intended for precise light dosimetry by continuously measuring the optical output power and adapting to the set parameter. Figure 3 shows optical feedback system design. Powerlux LED (1 W Power LED 620–640 nm,2–2,4 V, 350 mA / Violet, 405–410 nm, 3–3,2 V, 350 mA) was used. The measurement was performed with a powermeter and a spectrum analyzer (PM100 and C series spectrometer, Thorlabs, Germany, regularly calibrated by Innova (Thorlabs-Turkey Partner)). The systems calibrated separately for 405 and 635 nm with according to the center wavelength. The well plate can be changed easily as shown in Fig. 8. The system is equipped with optical power feedback mechanisms to ensure the precise delivery of optical power, as illustrated in Fig. 4. The core of the system’s design lies in its ability to provide continuous and accurate control over the optical power delivered to each well. To achieve this, a reflective lens is placed within the LED illumination probe’s optical path. This lens efficiently redirects a small fraction of the emitted light towards the integrated designed photodiode system. The photodiode is carefully chosen for its high sensitivity and response speed, allowing it to promptly detect even subtle changes in the optical output power. To process the signal received from the photodiode effectively, a transimpedance amplifier is employed. The transimpedance amplifier converts the current output of the photodiode into a voltage signal, amplifying it in the process. This conversion ensures that even minute variations in the light intensity are transformed into readily measurable electrical signals.

The system comprises a modular metallic case designed for the LED system, featuring interchangeable metallic heads that can be effortlessly adjusted to accommodate varying well sizes in different well plates. Because of its versatility, system can easily accommodate a wide array of experimental configurations, allowing researchers to quickly transition between various well plate sizes and kinds without requiring complicated setups.

#### Automated well plate illumination system

The LED probe was accurately positioned over the desired well location utilizing the precision three-axis movement system. Following the probe’s placement, the illumination was adjusted according to the user’s specific preferences. The system allows for the implementation of various light doses and illumination modes tailored to each individual well, providing researchers with the flexibility to customize their experimental conditions effectively. However, to conduct more effective experiments, the wells were utilized without completely filling each. This approach aimed to minimize the surface area exposed to light, thereby preventing light from affecting other wells containing experimental materials. By reducing the volume of liquid in each well, the potential for light dispersion across adjacent wells was minimized, thus ensuring that the experimental conditions remained isolated and unaffected by external factors. There isn’t a separate cooling mechanism for well-plate in the system. To confirm that the LEDs operate at constant temperatures, the system does, however, include LED light sources with cooling mechanism. Consequently, the well plates’ temperature cannot rise significantly while the LEDs are not producing heat, which reduces the possibility of diffusion-induced thermal effects [35].

#### User interface and optic power modes

The user interface was designed to allow users to select the proper light dose and illumination mode. The system has three modes to choose from: CW, pulse, and burst pulse. The modes were developed considering the application modalities indicated in the literature [23, 24]. The user can select the experiment duration and mode-specific parameters for each mode separately. Additionally, the well plate type and each well’s light mode characteristics can be chosen.

### Photodynamic therapy experiments

The experiments aimed to identify the most effective conditions for LED-based PDT treatment using 5-ALA, as well as to validate the system’s efficacy.

#### Cell culture

The HT-29 colon cancer cells and WI-38 lung fibroblast cells were cultured in Dulbecco’s Modified Eagle Medium (DMEM) supplemented with 10% fetal bovine serum (FBS), 1% L-glutamine, and penicillin in 25 cm2 plastic tissue culture dishes. The cells were then incubated at 37 °C.5-Aminolevulinic acid hydrochloride (5-ALA) (A7793 from Sigma Aldrich, Merck KGaA) was dissolved in sterile distilled water and stored in the dark. Various photosensitizer (PS) solutions were prepared for each experiment by diluting the stock solution with serum-free DMEM medium. Serum-free media was used to prevent cells from releasing porphyrin.

#### Determination of incubation time of 5-ALA

In a 96-well cell culture plate, 20,000 HT-29 and 20,000 WI-38 cells were seeded, and then the cells were incubated for 24 h at 37 °C. Following incubation, the cells were exposed to 0.5, 1 and 1.5 mM 5-ALA for 1–12 h at 37 °C. After treatment, cells were washed twice with PBS and lysed in 1% SDS solution to evaluate intracellular PpIX accumulation. PpIX deposition was measured using a SPECTROstar Nano® Absorbance Plate Reader at 405 nm, PpIX’s absorbance value (BDAS, LLC, Lexington, KY, USA). The optimal time for PDT incubation was identified to be the period with the highest absorbance. The ideal incubation duration was determined by testing three different dosages.

#### Cytotoxic effect of 5-ALA without irradiation

In a 96-well cell culture plate, with 20,000 cells per well, HT-29 and WI-38 cells were grown. Cells were cultured for 5 h in serum-free DMEM media containing 5-ALA at various concentrations (0.5, 1, 1.5, 2, and 3 mM). Cell survival activities were determined using MTT assays (Sigma-Aldrich, MO, USA). After incubating the cells for 24 h, the medium was aspirated and MTT solution was added. Following a further 4-hour incubation period at 37 °C, the medium was then removed. In the final step, remnant formazan crystals was removed byusing dimethyl sulfoxide (DMSO; Sigma-Aldrich; MO; USA). Using the SPECTROstar Nano® Absorbance Plate Reader, absorbance was measured at 490 nm(BDAS, LLC, Lexington, KY, USA).

#### Photoxicity of light

In HT-29 cells, the phototoxic impact of LED light was examined. Following a 5-ALA incubation period of 5 h, cells were exposed to radiation doses of 5, 10, 15 and 20 J/cm^2^ at 405 and 635 nm wavelengths alone or in combination. Control group was not exposed to the light. Fresh DMEM media containing 10% FBS were added to each well before exposing the cells to radiation. The cells were incubated for 24 h at 37 °C. The MTT assay was used to determine the phototoxicity.

#### In vitro photodynamic therapy

20.000 HT-29 and WI-38 cells were seeded in 96-well plates. According to the MTT results, the medium containing the 1-mM 5-ALA was replaced with fresh medium after the 5-hour treatment period. Three different types of LEDs were used to irradiate the cells, with a determined fluence rate of 30 mW/cm^2^ and radiant exposure of 5 J/ cm^2^. There was no administration of ALA or LED radiation for the control group. Cell viability was assessed by MTT assay after a 24 h incubation period. SPECTRO star Nano® Absorbance Plate Reader was used to measure sample absorbances (BDAS, LLC, Lexington, KY, USA). The irradiance was given both separately and simultaneously during the experiments. When given separately, each wavelength (405 nm and 635 nm) was delivered independently with the specified irradiance values. However, when given simultaneously, the power was equally divided between the two wavelengths. For example, a total radiant exposure of 20 J/cm2 was delivered as follows: 10 J/cm^2^ at 405 nm and 10 J/cm^2^ at 635 nm. The combination of these two wavelengths was used to assess their effects on the cells.

## Results and discussion

To ensure the stability of radiation and the successful completion of PDT treatment, the MAWPLIS output was adjusted based on voltage (V), current (A), power (P), and temperature (0 °C). The optimal balance of wavelength (nm) and optical power (W) at 635 nm was maintained to achieve this objective. The individual parameters were systematically altered while maintaining constant levels for the other factors, as enumerated below.

### Electrical Control Parameters for Laser Diodes and Temperature Stability

Experimental measurements of the current variation as a function of voltage and relative LED power are presented in Fig. 2. The PID control algorithm analyzes the feedback and target values continuously in relation to the obtained voltage and current. To ensure that the values remain within the predetermined 1% error rate, the voltage drop is continually monitored across the aluminum resistance connected in series with the laser diode’s internal resistance. The system’s temperature stability is critical to obtaining the reliable results. PDT laser systems often experience significant problems with optical power stability at low output power levels and wavelength stability at high output power levels. To prevent oscillations in optical power and fluctuations in the wavelength peak at different powers, the current and voltage control circuitry of the TEC system must function precisely. Figure 4 illustrates the impact of temperature on LED wavelength.

**Fig. 5:**
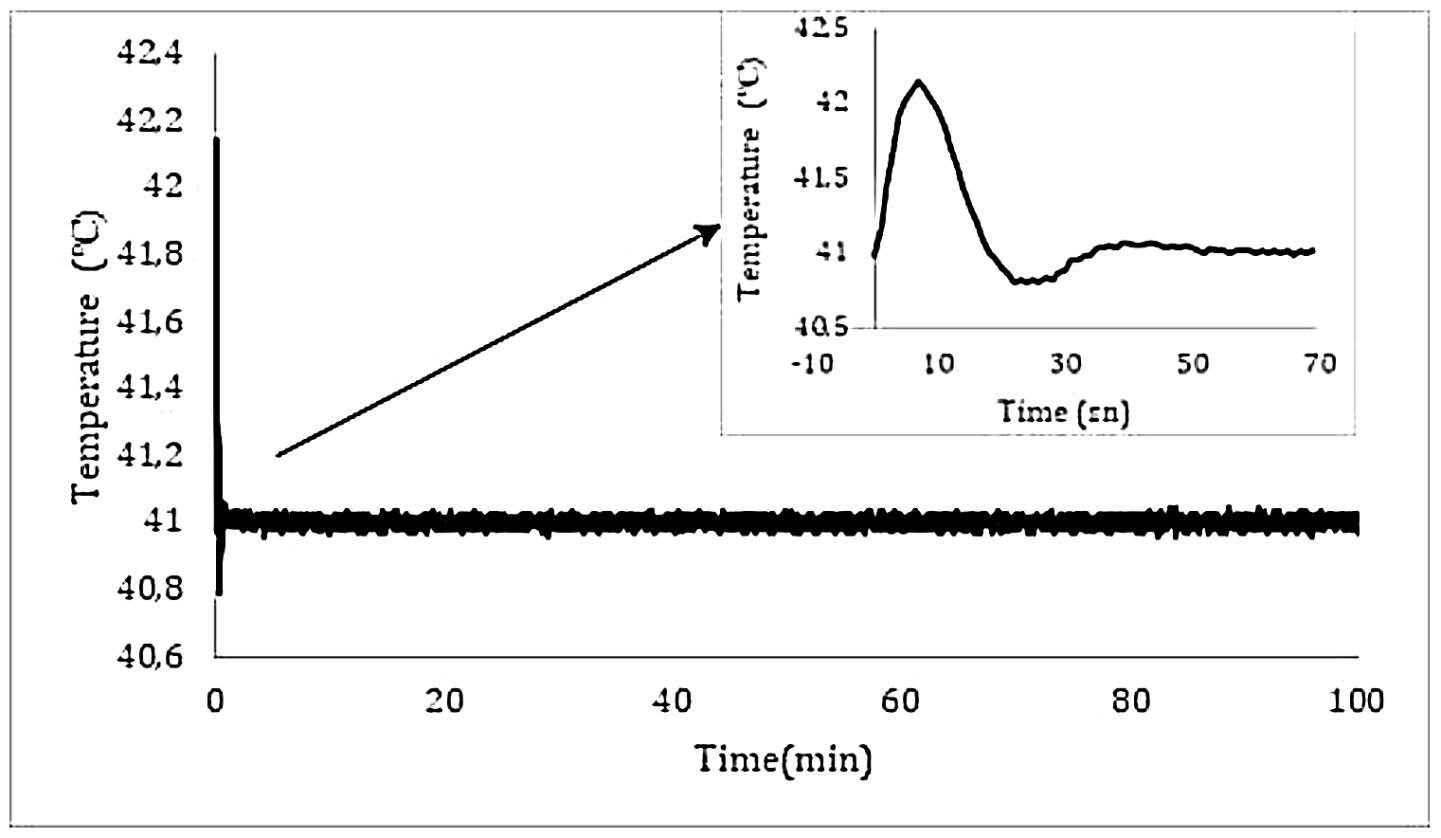
**a**) The overall temperature stability and the system’s early response to radiation are shown. The inset graph depicts the system’s response over the first 70 s, with temperature stabilization occurring after 40 s

A long-term temperature stability graph was generated based on values that were monitored over a period of six hours, as illustrated in Fig. 5a. Figure 5b shows the temperature stability over a shorter time frame. During the experiment, the LED temperature was held constant at 15 ± 0.2^0^C degrees, while the current was varied from 0 to 4 A, which represents the maximum change in current for radiation. The measured wavelength, the peak wavelength of the LED emission, was demonstrated in Fig. 4 (Wavelength was measured with spectrophotometer). After the initial radiation, deviation become stabilized within 20 s and returned to its pre-illumination values after 20 s. The MAWPLIS system was calibrated for all radiation modes after the temperature was maintained within a 0.2^0^C error range. The system met these requirements for all radiation modes, with a temperature stabilization time of 5 s at room temperature.

### Radiation modes with wavelength and optical power stability

The device’s maximal clinical working time is often expected to be 90 min, and data was collected over a period of 6 to 24 h to determine the system’s stability limits. The suggested system’s optical power stability was tested to be within 1% error between 0 and 500 mW, the spectrum stability was measured to be 635 ± 1 nm, the overall system temperature stability was measured to be 44 ± 0.2^0^C. A graph depicting the long-term temperature stability was checked by monitoring the values for six hours, as demonstrated in Fig. 5. Inset shows the temperature stability over a shorter period of time (0 to 70 s). During the experiment, the LED temperature was kept at 15 ± 0.2 °C, while the current was altered from 0 to 4 A, which corresponds to the current change for minimum and maximum radiation. The MAWPLIS system was calibrated for all radiation modes once the temperature was maintained within a 0.2 ^0^C error range. The temperature stabilization time was determined to be 5 s at room temperature, and the system met these standards for all radiation modes.

**Fig. 6:**
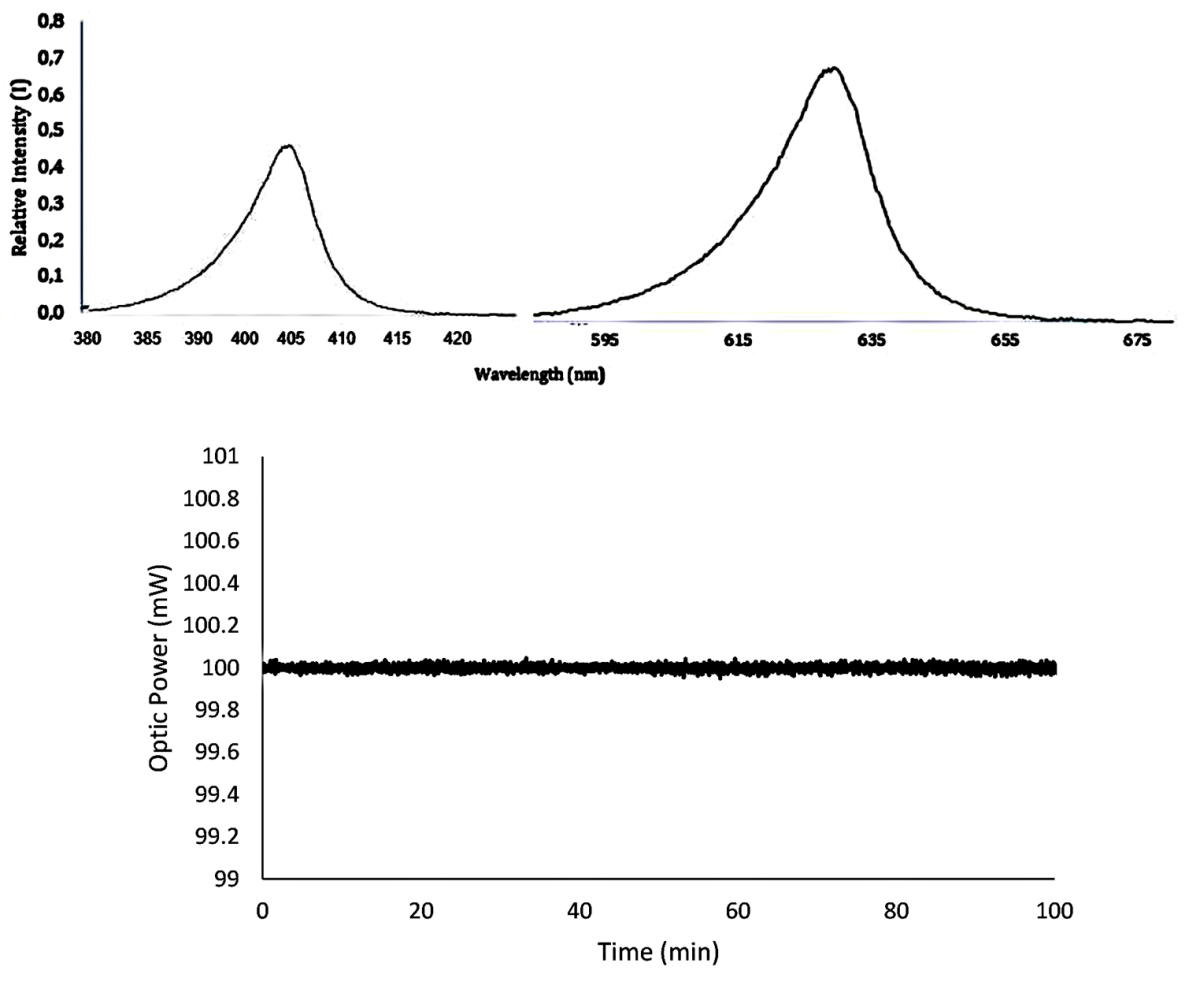
Radiation mode, (**a**) The wavelength spectrum stability over time, and (**b**) Optic power output stability of wavelength throughout a 100-minute test period at 500 mW

Figure 6 depicts critical insights into the stability of the radiation mode in the Multi-mode Automatized Well-plate PDT LED Laboratory Irradiation System (MAWPLIS). Two significant features can be depicted from the figure: (1) the wavelength spectrum stability over time, and (2) the current and output stability of the wavelength during a 100-minute time interval at 500 mW. The wavelength spectrum stability graph demonstrates the MAWPLIS system’s remarkable capability to maintain consistent and precise emission wavelengths throughout the testing period. This exemplary stability is essential for ensuring accurate and reliable light dosimetry during photodynamic therapy (PDT) investigations, as any fluctuations in the emitted wavelengths could significantly impact treatment outcomes. Additionally, the current and output stability graph demonstrates the MAWPLIS system’s exceptional performance under continuous operation at 500 mW. The system consistently maintains both the current and output levels, reaffirming its reliability and suitability for extended PDT experiments. The findings presented in Fig. 6 validate the MAWPLIS system’s reliability and robustness, emphasizing its significance in advancing PDT research and optimizing therapeutic outcomes.

**Fig. 7:**
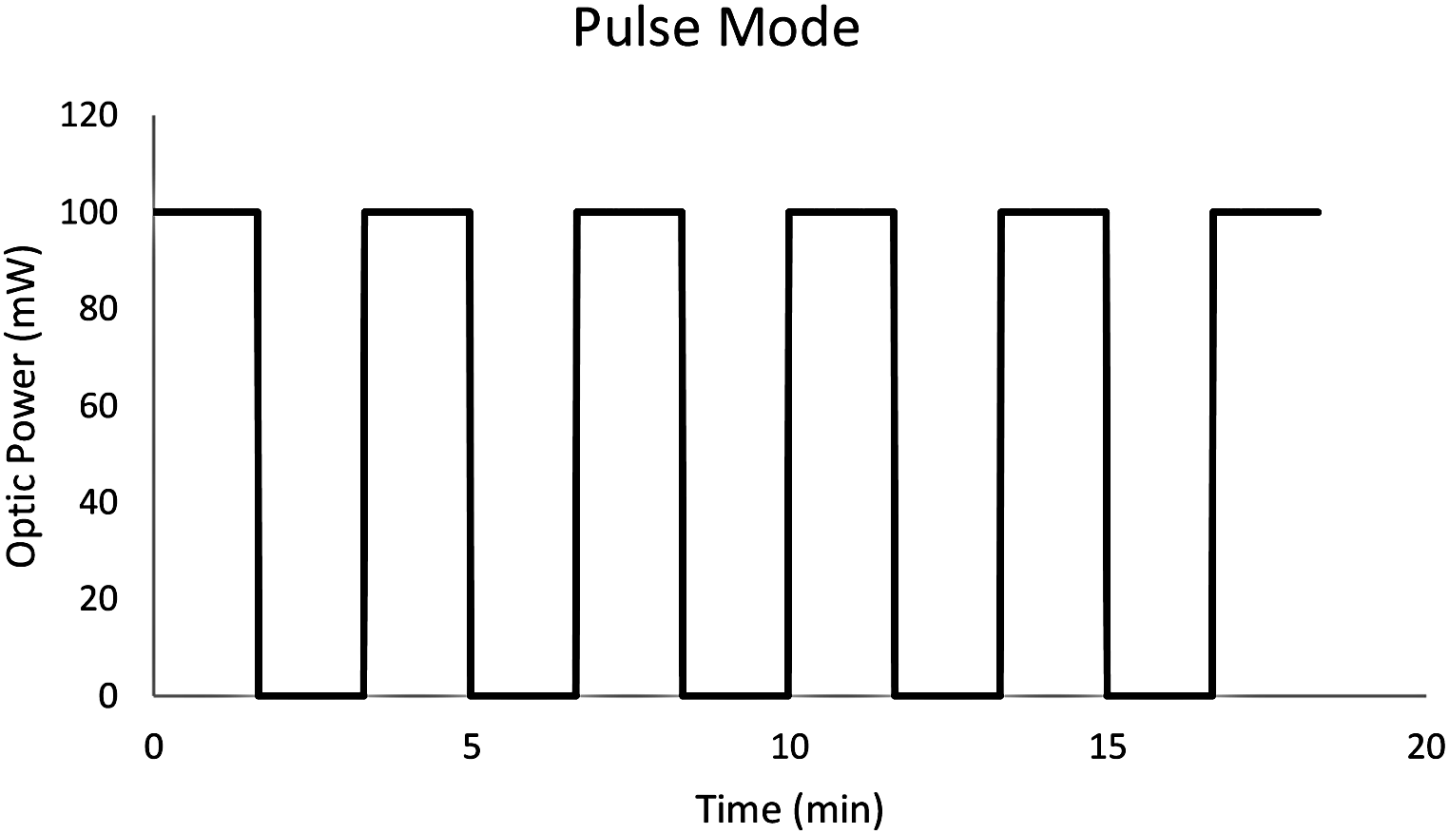
MAWPLIS system for pulsed mode

Figure 7 showcases the Multi-mode Automatized Well-plate PDT LED Laboratory Irradiation System (MAWPLIS) in operation with pulsed mode. The figure illustrates the versatile capabilities of the MAWPLIS system, which is equipped with three distinct radiation modes, each designed to deliver specific light doses to the target cells. The different radiation modes provide researchers with a powerful tool for assessing various light dosages and photosensitizer concentrations, enabling the optimization of PDT protocols for enhanced therapeutic efficacy. The flexibility and automation offered by the MAWPLIS system streamline PDT investigations, significantly reducing user errors and ensuring precise and reproducible results. The system’s capability to deliver tailored radiation modes enhances its utility in investigating the phototoxic impact of PDT in diverse cellular contexts. Figure 7 emphasizes the MAWPLIS system’s versatility and potential in advancing PDT research, offering a promising avenue for the development and validation of PDT LED systems for more targeted and effective cancer therapies.

**Fig. 8:**
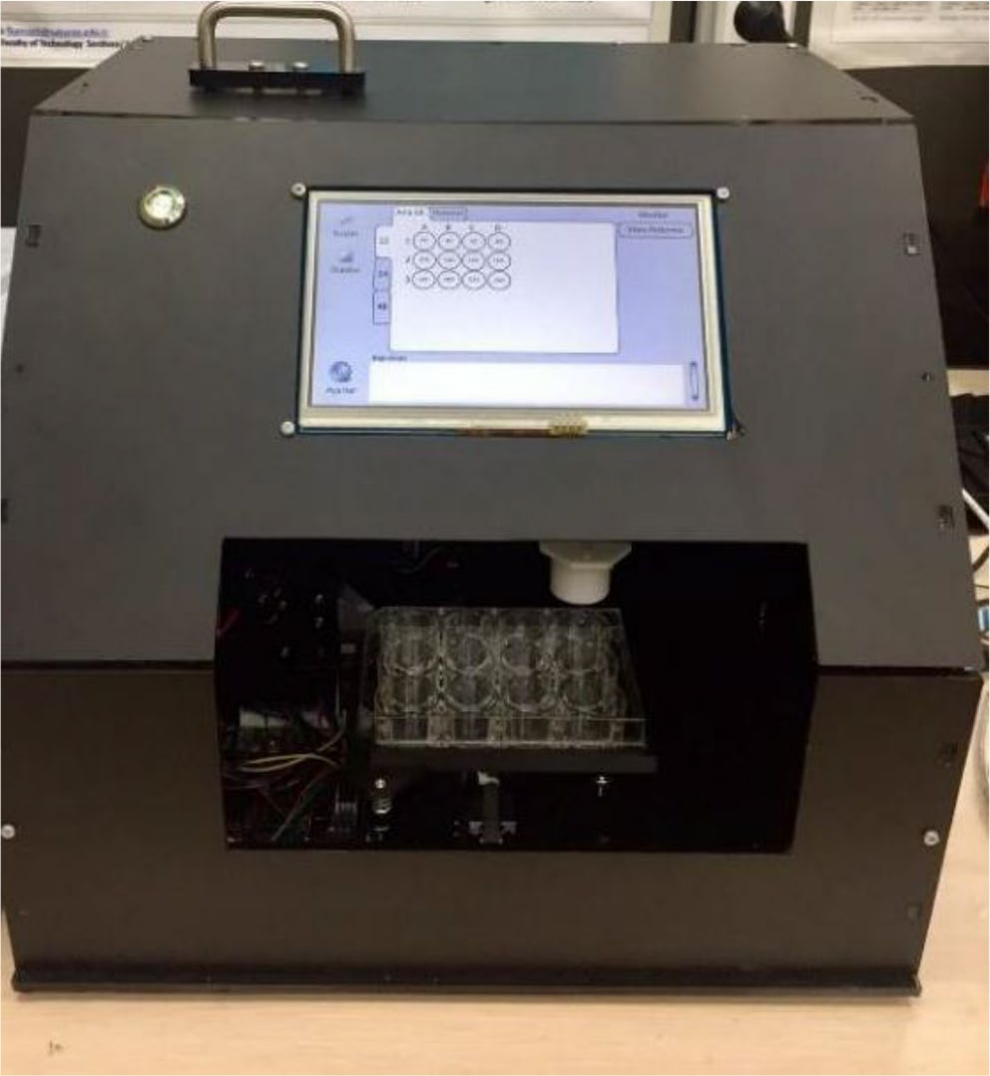
Mechanical design of 3D automatic LED system MAWPLIS

Figure 8 presents the mechanical design of the 3D automatic LED system, the Multi-mode Automatized Well-plate PDT LED Laboratory Irradiation System (MAWPLIS). This figure shows the innovative and robust engineering of the MAWPLIS system, which plays a pivotal role in automating and streamlining the PDT investigations. The mechanical design of MAWPLIS is specifically tailored to accommodate the well-plate format, enabling simultaneous and precise illumination of multiple cell samples.

The user interface’s design makes it simple to pick the optical power mode, optical power, time, and well-plate cell position. The system starts once all of the experimental data for all selected wells has been entered. The practitioner can observe each part of the experiment using the information screen (Table 1). The experiment time and time saving for automated experiment for each well on the well plate.

**Table 1:** The experiment time and time saving for automated experiment for each well on the well plate

Typical PDT Experiment Energy	CW time (sn)	Pulse Mode Time (sn)	Well Plate (12) Experiment Time (min)	Well Plate (96) Experiment Time (min)	Time Saving (With preparation)
1.5 J/cm^2^	50	100	10–20	80–160	1 h
3 J/cm^2^	100	200	20–40	160–320	2 h
6 J/cm^2^	200	400	40–80	320–640	3.5 h
9 J/cm^2^	300	600	80–160	640–1280	4.2 h
12 J/cm^2^	400	800	320–640	1280–3560	5.4 h

The pulse frequency of the LED irradiation in MAWPLIS was 1 Hz, with a pulse period of 1 s on and 1 s off. This indicates a duty cycle of 50%, where the LEDs were turned on for half of the time and turned off for the other half. The operator has the flexibility to choose any value for the irradiance according to the specific requirements of the experiments. In this study 1 Hz was selected for the pulse frequency of the LED irradiation (Operator can select the on duration and the off duration separately).

To improve the system’s automation and widen its possible uses, the Z-scan technology can be integrated. The Z-scan approach can be used to diagnose various cell types [36]. Furthermore, it allows for the monitoring of cellular damage after various treatment strategies, as demonstrated in the study by Ghader et al. [37]. This optical technology highlights its significance and potential impact on biomedical research and clinical practice.

### Cell culture experiment results

The proper incubation period is critical for increasing treatment efficacy since irradiation at the point of cellular saturation with the photosensitizer has a significant impact on treatment outcomes. The result relates to the uptake of 5-ALA by malignant and non-cancerous cells at different 5-ALA concentrations and incubation periods. Our findings indicate that 5-ALA absorption in HT-29 cells is extremely poor during the first few hours of incubation. Nevertheless, 5-ALA absorption peaked after 5 h of incubation and then steadily dropped over the course of the following hours. On the other hand, WI-38 cells had lower absorbance values because more PpIX is accumulated in cancer cells than in healthy cells.Moreover, the intracellular PpIX in WI-38 cells was almost completely removed within 4 h.

These findings led to the selection of a 5-hour incubation period for 5-ALA (4–6) in HT-29 cells for further analysis. Our findings were consistent with previous experimental studies on 5-ALA in HT-29 cells [4, 5]. However, different incubation times have been observed for effective 5-ALA absorption in different cells [6, 7, 38]. This suggests that the cell type affects the cellular uptake of ALA.

**Fig. 9:**
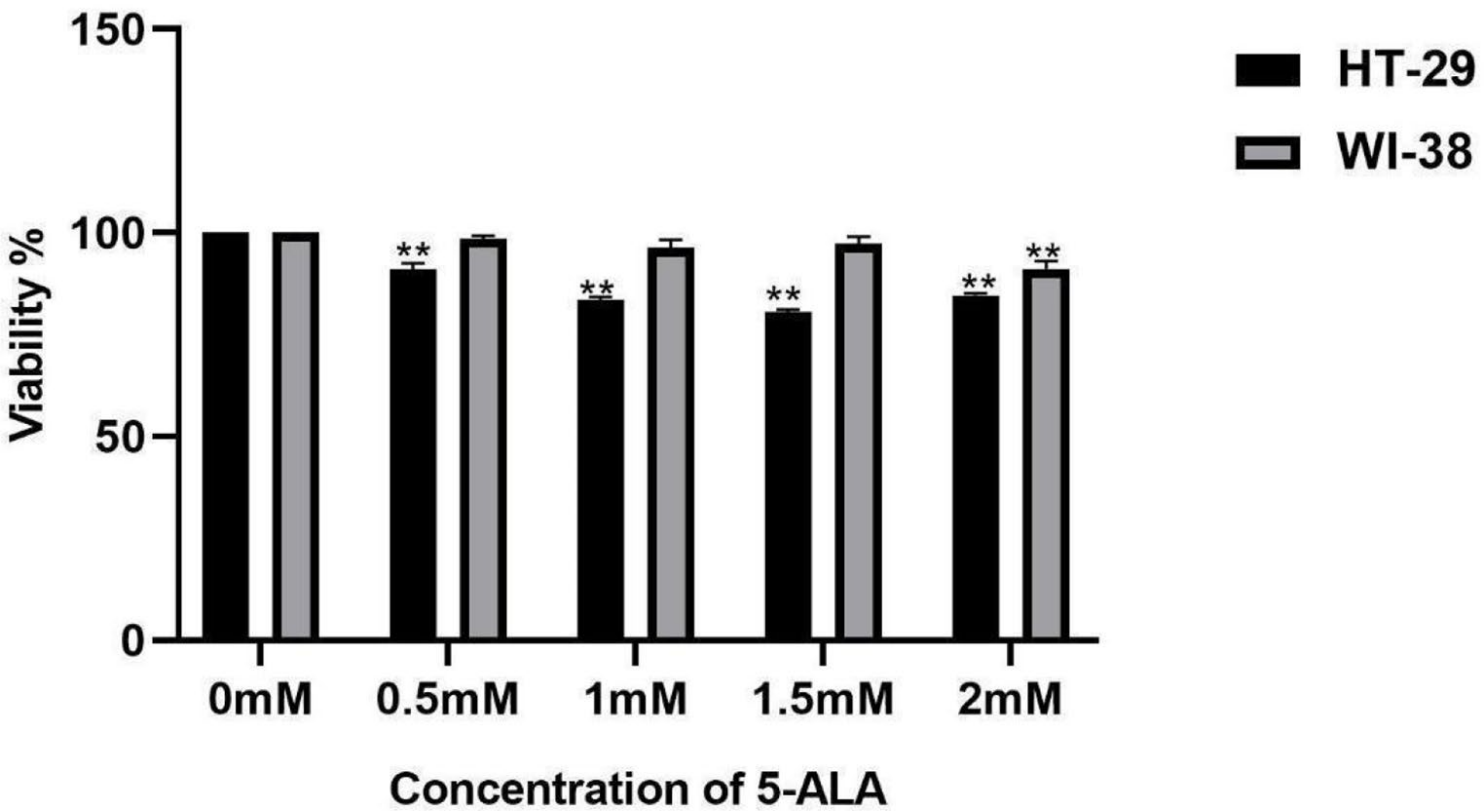
Viability of HT-29 and WI-38 cell lines following treatment with 5-ALA without irradiation (Anova *p* < 0.05)

In Fig. 9, the effects of increased concentrations of 5-ALA on the viability of HT-29 and WI-38 cells’ without irradiation was analyzed by MTT analysis. No significant cytotoxic effect was observed (*p* < 0.05). Because the healthy cell line WI-38 accumulated PpIX at a lower rate than cancer cells, the toxicity of 5-ALA was decreased. Higher 5-ALA concentrations, on the other hand, caused toxicity in HT-29 cells. These outcomes could be attributed to 5-ALA’s chemical characteristics, which make HT-29 cells more viable.

**Fig. 10:**
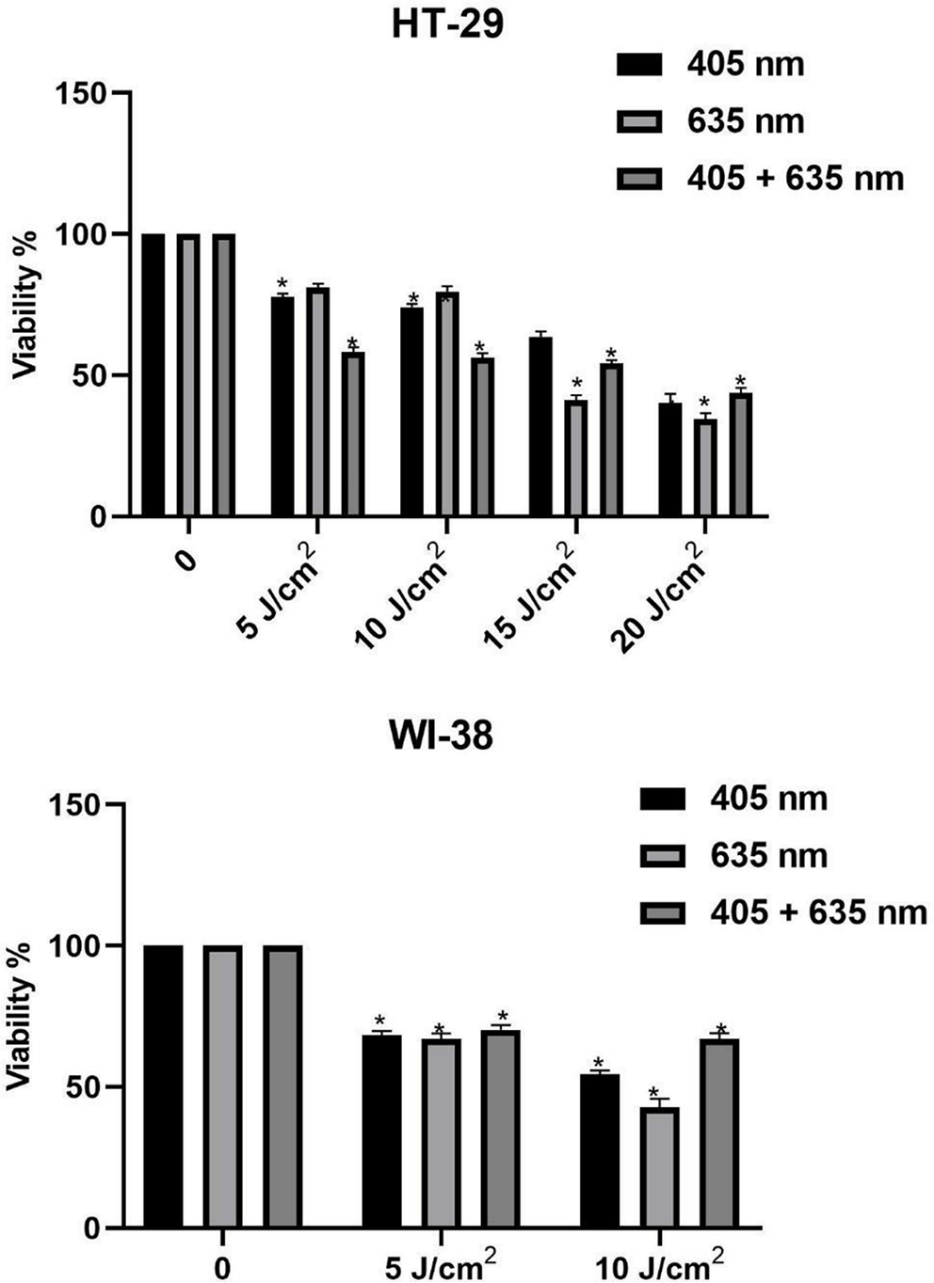
Cellular viability of 1 mM 5-ALA treated HT-29 and WI-38 cells following different radiant exposures for different wavelengths.(*lower error from previous experiments [39]). Lower viability is aimed for the experiment

The effects of wavelengths (405 nm, 635 nm, and combined 405 nm + 605 nm) on HT-29 cells were examined in the context of photodynamic therapy within the settings of our study as seen in Fig. 10. Results showed that HT-29 cell viability significantly decreased after irradiation, and that this reduction depended on the dosage used. Remarkably, 405 nm + 635 nm was found to be the most efficient application at 5 J/cm2 and 10 J/cm2 fluence rates. It was also evident that using lower light doses (5 and 10 J/cm^2^) was more favorable in preventing necrotic cell death, countering the detrimental effects of intense light during 5-ALA-PDT on HT-29 cells. It’s important to note that experiments were conducted in petri dishes, minimizing the influence of penetration depth.

Further investigation into the absorption features of Protoporphyrin IX (PpIX) has shown unpredicted findings. Despite its higher absorption coefficient, the 405 nm wavelength had difficulty permeating the skin effectively. In contrast, the 635 nm wavelength, despite having a lower absorption coefficient, has emerged as a popular choice in 5-ALA-PDT studies due to its greater skin penetrating properties. Notably, Tomoya Hatakeyama et al. [40] showed that blue light outperformed red light in terms of efficacy, confounding common notions about light penetration depth in vivo. Interestingly, the combined 405 nm and 635 nm wavelengths showed reduced harm to WI-38 cells, while proving more effective for HT-29 cells compared to either wavelength alone.

Results highlighted the robust synergy between the 405 nm and 635 nm wavelengths on HT-29 cells following exposure to 1 mM 5-ALA for 5 h. This suggests that the optimal combination of medication and low doses, alongside precise irradiation timing, could maximize the impact on cancer cells during PDT. Additionally, the integration of the MAWPLIS system, with its automated adjustments, notably reduced the experimental duration by 30–55% across various applications, ensuring consistent and stable trial parameters.

In the field of Photodynamic Therapy (PDT), a peculiar anomaly has emerged concerning the disparate outcomes of irradiation at 405 nm and 635 nm wavelengths, despite the significantly higher absorption coefficient of Protoporphyrin IX (PpIX) at 405 nm.“. This observation prompts a nuanced exploration of the intricate mechanisms that govern the interplay between light wavelength and the efficacy of photosensitizers. Our revised examination aims to untangle this puzzle, venturing into multifaceted considerations ranging from the physical properties of PpIX to the responses within cells. This journey culminates in a quantitative comparison with prior in vitro studies, enriching the contextual understanding of the observed outcomes within the dynamic landscape of PDT. As we traverse this investigative path, we contribute to the growing knowledge that forms the foundation of Photodynamic Therapy, shedding light on the complexities that underscore its therapeutic potential.

## Conclusion

In the realm of cancer therapy, photodynamic therapy (PDT) stands out as a promising approach, leveraging photosensitizers and light sources to selectively target malignant cells. Laser light sources, with their coherent and collimated output, have shown superior efficacy in PDT. However, the complex and costly nature of laser systems restricts their use in well-plate irradiation setups. To address this limitation, LED-based PDT systems have emerged as a viable alternative, offering cost-effectiveness and ease of use. Nonetheless, existing LED systems have been hindered by their dependence on manufacturer-provided optic characterization, neglecting critical experimental parameters like temperature and current that impact optic stability.

This study sought to design an LED system with stable output characteristics, mitigating user errors, and enabling PDT-based cell line investigations in well-plate environments. The pursuit of a sterile environment without manual handling was a key consideration in the system’s development. By incorporating temperature and current control parameters, the LED system achieved consistent and precise light dosing, surpassing previous systems’ limitations.

The results of this study demonstrated the LED system’s capacity to deliver the necessary light dose to cells effectively, markedly reducing user error compared to conventional setups. Notably, the LED output stability was markedly enhanced due to the integrated temperature and current control measures. The custom well-plate holder facilitated sterile and convenient cell studies, eliminating contamination concerns.

The successful implementation of the LED system opens a new chapter in PDT-based cell line studies within well-plate environments. The capability to perform experiments in a sterile setting without manual handling assumes heightened significance in the present age of infection control. Moreover, the stable and consistent LED output makes it an alluring choice compared to costly and intricate laser systems. This LED system, characterized by its affordability and reproducibility, holds immense value for researchers engaged in PDT-based cell line studies.

In conclusion, the designed LED system presents an impactful and cost-effective alternative to conventional laser-based PDT setups for well-plate irradiation systems. The system’s incorporation of temperature and current control parameters ensures a dependable and steady output, while the custom well-plate holder streamlines experimentation in a sterile environment. The LED system’s versatility paves the way for future research to explore the effects of different LED wavelengths and power densities on cell viability and PDT effectiveness. Additionally, its integration with other therapeutic modalities could unlock novel and synergistic treatment approaches, ultimately enhancing the prospects of successful PDT outcomes in cancer therapy.
